# Outcome evaluation of Active Herts: A community-based physical activity programme for inactive adults at risk of cardiovascular disease and/or low mental wellbeing

**DOI:** 10.3389/fpubh.2022.903109

**Published:** 2022-09-09

**Authors:** Angel M. Chater, Joerg Schulz, Andy Jones, Amanda Burke, Shelby Carr, Dora Kukucska, Nick Troop, Daksha Trivedi, Neil Howlett

**Affiliations:** ^1^Institute for Sport and Physical Activity Research, University of Bedfordshire, Bedford, United Kingdom; ^2^Centre for Behaviour Change, University College London, London, United Kingdom; ^3^Department of Psychology, Sports, and Geography, University of Hertfordshire, Hatfield, United Kingdom; ^4^Norwich Medical School, University of East Anglia, Norwich, United Kingdom; ^5^School of Psychology, University of Plymouth, Plymouth, United Kingdom; ^6^Centre for Research in Public Health and Community Care, University of Hertfordshire, Hatfield, United Kingdom

**Keywords:** physical activity, inactivity, exercise, cardiovascular risk, mental wellbeing, COM-B, motivational interviewing, behavior change intervention

## Abstract

**Background:**

A high proportion of UK adults are inactive, which can lead to a range of physical and mental health concerns. Active Herts is a community-based physical activity programme for inactive adults at risk of cardiovascular disease and/or low mental wellbeing. This paper provides a pragmatic evaluation of this programme.

**Method:**

This longitudinal study observed 717 adults (68% female, mean age = 56.9 years) from the “Active Herts” programme. Programme users were provided with a 45-min consultation with a “Get Active Specialist,” who talked them through an Active Herts self-help booklet and then signposted them to free or subsidized local exercise sessions. Programme users were followed up with a booster call 2 weeks later. The Get Active Specialist was a registered exercise professional (REPS Level 3), with additional training from the study team in motivational interviewing, health coaching, COM-B behavioral diagnosis and delivery of behavior change techniques (BCTs) in practice. The Active Herts booklet contained theoretically-driven and evidence-based BCTs to translate behavioral science into public health practice. Physical activity (Metabolic Equivalent Time [METs], measured using the International Physical Activity Questionnaire (IPAQ), perceived health (EQ-5D-5L) and mental wellbeing (Warwick-Edinburgh Mental Wellbeing Scale: WEMWBS) were measured at baseline, 3, 6 and 12 months.

**Results:**

At the end of the 12-month programme, users showed sustained improvements in physical activity (by +1331 METS), exceeding weekly recommendations. Sitting (reducing by over an hour per day), sporting participation, and perceptions of health were also improved, with improvements in mental wellbeing in the first 3 months.

**Conclusion:**

Designing and delivering a community-based physical activity programme that is theoretically-driven and evidence-based with frequent behavior change training and supervision can yield a significant increase in self-reported physical activity, reduction in sitting behavior and improvements to perceived health and mental wellbeing. Future research should extend this approach, utilizing a real-world, pragmatic evaluation.

**Trial registration:**

ClinicalTrials.gov, identifier (NCT number): NCT03153098.

## Introduction

Physical inactivity is the fourth leading risk factor for mortality worldwide, and is related to one in six UK deaths (1). The World Health Organization (2) and UK Chief Medical Officer (3) recommend that adults should perform 150 min of moderate to vigorous physical activity (MVPA) or 75 min of vigorous physical activity (VPA) per week. Physical activity should be spread across the week and can be gained in bouts of 10 min or more. In addition, muscle strengthening activities, such as lifting weights or heavy shopping bags, are recommended on at least 2 days each week. In Sport England's Active Lives Adult survey (4), 62% of men and 60% of women were considered as “active,” categorized as meeting at least 150 min of combined MVPA. Physical activity follows a social gradient with adults living in lower socio-economic areas found to be less likely to participate in the recommended MVPA and walking (5).

Sport England (4) defines adults who are insufficiently active in two categories; fairly active (30–149 min of weekly combined MVPA), representing 11.6% of the population (5.3 million); and inactive (<30 min of MVPA per week), representing 27.5% of the population (12.5 million). Sedentary behavior, defined as any waking activity, such as sitting, reclining, or lying which expends <1.5 metabolic equivalents (6), has been identified as an independent risk factor for poor cardiometabolic health that should be considered in addition to physical inactivity (7). Figures for sedentary behavior show that approximately a third of the adult population (29%) in England, spend on average 6 or more hours of weekday time sedentary (e.g., watching TV, reading and computer use). Men and woman respectively spend on average 4.8 hours and 4.6 h per weekday and 5.3 h and 4.9 h per weekend day being sedentary (8).

Meeting the recommended physical activity guidelines and breaking up sitting can help to reduce the risk of long-term conditions such as, obesity, type 2 diabetes, cardiovascular disease, stroke, cancer, and poor mental health while also being beneficial to those living with these conditions (2, 3, 9, 10). It can further improve subjective wellbeing, mental health and quality of life (11, 12). Individuals living with cardiovascular disease (CVD) and a combination of CVD and type 2 diabetes report lower levels of physical activity and greater sedentary behavior (13). Engagement in physical activity should be encouraged in clinical practice and is the focus of the Sport England “WeAreUndefeatable” campaign (14), which targets those with long-term conditions. People who are inactive, living in areas of deprivation and/or with medical conditions putting them at risk of CVD, such as hypertension, obesity, diabetes or lowered mental wellbeing are, therefore, an important target group for physical activity and sedentary behavior interventions.

The Behavior Change Wheel (BCW) (15, 16) offers a layered framework to intervention development, with the COM-B model at the hub of the wheel. This model postulates that behavioral performance is influenced by an individual's capability, opportunity, and motivation. These COM factors can explain a large amount of variance in physical activity (17) and sitting behavior (18), highlighting psychological capability (such as action planning, behavioral regulation to overcome habits and self-monitoring) and reflective motivation (such as intentions, self-efficacy, and exercise self-identity) as key drivers. Interventions with inactive adults show small to moderate effect sizes post-intervention, and small but statistically significant effects 6 months or more after intervention-end (19). Coding interventions using the Behavior Change Technique (BCT) Taxonomy Version 1 (20), a systematic review showed that the BCTs associated with effective physical activity interventions that may increase the likelihood of significant and sustained behavior change include: “action planning,” “instruction on how to perform the behavior,” “prompts/cues,” “behavior practice/rehearsal,” “graded tasks,” and “self-reward” (19). The delivery of such BCTs should use effective communication styles, addressing capability, opportunity, and motivation, alongside ambivalence and intrinsic motivation (21, 22). Motivational interviewing (23) has been shown to be an effective communication style to change several health behaviors including physical activity (24), with modest improvements in physical activity found in those living with long-term health conditions (25). In combination, motivational interviewing (23) and health coaching (26) can be used to evaluate and elevate an individual's Capability (physical and psychological), Opportunity (social and physical), and Motivation (reflective and automatic) to engage in behaviors such as physical activity, and effectively deliver BCTs to enable change (27, 28).

The extent to which physical activity behavior change interventions are effective for the intended target population as opposed to being effective just for those participating in the intervention, remains contentious (29). Whilst highly controlled trials can provide evidence for the efficacy of an intervention under ideal conditions, pragmatic field studies enable an evaluation of their effectiveness in real world conditions. “Active Herts” is an evidence-informed, theoretically-driven, community-based physical activity programme designed to support inactive adults with elevated risk of CVD and/or low mental wellbeing living in four areas of deprivation in Hertfordshire (Herts) (30). The programme was developed using the Behavior Change Wheel, with BCTs selected based on previous evidence of what has worked to sustain physical activity behavior change (19), mapped to the COM-B (30). The programme was implemented in a real-world setting, offered as part of routine care, with a parallel pragmatic observational research design and process evaluation (28). This paper provides an evaluation of this pragmatic real-world intervention programme, built on health psychology and behavioral science.

## Methods

### Design

A prospective longitudinal observational design was employed, with planned comparisons of the outcome measures to estimate change from baseline to 3, 6, and 12 months. There were two types of delivery planned. The “standard delivery” (Stevenage and Hertsmere) signposted programme users to local physical activity opportunities, while the “enhanced delivery” (Watford and Broxbourne) provided free access to tailored exercise classes with additional support from exercise buddies. Active Herts received initial funding from Sport England and local authority resources (e.g., gym space), and the original programme was delivered between November 2015 and December 2018. This evaluation is reported according to the Transparent Reporting of Evaluations with Non-randomized Designs (TREND) guidelines (31). The programme was registered**:** ClinicalTrials.gov identifier (NCT number): NCT03153098 and a protocol has been published (30). The full Active Herts package can be found on the Local Government Association website (32). More details of the programme can be found on the Active Herts website (33).

### Participants

#### Eligibility

The Active Herts programme was available to Hertfordshire residents *via* referral from a general practitioner (GP) or self-referral. The programme was aimed at adults (over 16 years of age) who were inactive (achieving <30 min of MVPA per week), and who had one or more risk factors for CVD and/or low mental wellbeing, as defined by the referrer. Risk factors for CVD included: diabetes, hypertension, high cholesterol, obesity (BMI > 30 or BMI > 28 if one or more co-morbidities), and/or smoking.

#### Recruitment

The programme deliverers, referred to as “Get Active Specialists,” were Registered Exercise Professionals (REPs) and responsible for the recruitment of willing programme users to this evaluation and data collection. Active Herts programme users were recruited through 23 GP services: five in Broxbourne; five in Hertsmere; six in Stevenage; four in Watford.

### Programme materials and procedure

Following referral, contact with programme users was managed using ReferAll software (34). Programme users in both delivery groups received an initial 45–60 min consultation with a Get Active Specialist (with additional consultations at 3, 6, and 12 months), an Active Herts booklet, a booster call at 2-weeks, as well as information about and access to physical activity opportunities in their local area. Programme users in the enhanced delivery group were in addition also offered 12 weeks of free exercise sessions developed specifically for the Active Herts programme and often run by the Get Active Specialist. The enhanced delivery group also included a “buddy” system where they could be paired with a volunteer buddy to help them attend the exercise classes and provide emotional support if needed. Programme users in the standard delivery group were signposted to pre-existing physical activity sessions (e.g., swimming, walking, and football), which were often discounted but not planned to be subsidized from Active Herts. Aside from access to the range of free or discounted group activity sessions over the first 12 weeks, there were no additional incentives for programme users to attend consultations.

The Active Herts programme included BCTs targeting all six components of the COM-B model and were previously found in effective physical activity interventions in a synthesis of 26 studies (19). BCTs (e.g., “*1.1. Goal setting (behavior)*; “*1.2. Problem solving*;” “*9.2. Pros and cons*;” “*1.4. Action planning*;” “*3.1 Social support (unspecified)*;” “*7.1. Prompts/cues*;” “*4.1. Instruction on how to perform behavior*;” “*8.7. Graded tasks*;” “*6.1. Demonstration of the behavior*;” “*8.1. Behavior practice/rehearsal*;” “*10.9 Self-reward*”), were included in the booklet given to programme users, in marketing materials, in the 45–60 min consultation by the Get Active Specialists, and in the exercise classes. Supplementary Table 1 provides a detailed breakdown of the BCTs and theoretical basis for each phase of the programme, and full details are available in the published protocol (30).

#### Get Active Specialists

A Get Active Specialist was employed by a local organization (e.g., borough council, leisure provider, and football club) in each of the four Hertfordshire localities and had a minimum of level 3 Register of Exercise Professionals (REPs) and GP Exercise Referral qualifications. A 2-day workshop was developed and led by AC (21), supported by NH. This covered how to identify barriers and facilitators to physical activity in relation to capability, opportunity, and motivation to create a COM-B behavioral diagnosis (21, 22, 35). This was facilitated with the communication style motivational interviewing (23, 36), and the GROW model of health coaching (GROW: goal, reality, options, will/way forward) (26). This training (21) followed the “engage, focus, evoke, and plan” strategy of motivational interviewing, with an emphasis on the consultation allowing partnership, acceptance, compassion and evocation (PACE) (37).

Core communication skills to support an effective consultation (35, 38) namely the RULE (Resist the righting reflex; Understand client motivation; Listen; Empower), OARS (Open-ended questions, Affirmations, Reflective listening, Summaries), empathy, rapport and rolling with resistance were covered in the training, and linked to the delivery of the BCTs (30). In addition, the GROW model (26) helped guide the Get Active Specialist through the consultation process, encouraging four segments of the conversation which focused on the client's goals, reality, options and will/way forward. This enabled conversations that supported programme users to take ownership of developing their own goals, overcoming barriers, specifying plans, and rewarding themselves for progress.

The training consisted of educational elements in relation to the BCW, motivational interviewing and health coaching, worked examples, a detailed overview of the BCTs included in the Active Herts materials and how they linked to the COM-B and wider BCW, and role play exercises that were recorded for reflection. To monitor consultations and maintain programme fidelity, the Get Active Specialists and project lead from the host Active Partnership met for booster sessions with AC and NH every 3 months throughout the duration of the programme. The booster sessions offered supervision, a re-cap on knowledge, and supported continued skill development through role plays and self-reflection. Get Active Specialists would bring pre-recorded client consultations (anonymized and with the permission of the client) to these booster sessions, which would be reviewed by AC and NH prior to the sessions for feedback and with the full team in the booster sessions, to evaluate whether consultations were MI-congruent and appropriate BCTs were being used. The team would listen and identity the barriers discussed with the client, highlighting them as “missing links” in the COM-B analysis to target, alongside “hooks” that highlighted desire for change that could be used to anchor the conversation. These booster sessions provided a safe space for the specialists to discuss any barriers to successful delivery of the programme, reflect on their consultation skills, for the team to give feedback on content and check the fidelity of the delivery of the programme, and to highlight what was working well and what might need to change. This process was seen as one of the strengths to the programme as highlighted in the externally-led process evaluation (28).

### Active Herts programme outcome measures

#### Primary outcome measures

Physical activity and sitting time were measured with the International Physical Activity Questionnaire (39). Two questions asked about the number of days and daily time spent participating in moderate and vigorous physical activity, and walking respectively, with the minimum being 10 min at a time. A metabolic equivalent of task (MET) score was then calculated for each activity type by weighting its energy requirements, with 3.3 METs for walking, 4 METs for moderate-intensity activity, and 8 METs for vigorous-intensity activity. A total activity MET score was then calculated by adding these weighted task scores (40). A single item asked about the amount of time spent sitting on an average weekday over the last week. Two additional questions asked about the amount of time spent doing sports and on how many days a week.

#### Secondary outcome measures

Psychological wellbeing was measured using the Warwick-Edinburgh Mental Wellbeing Scale (WEMWBS) (41), a 14-item scale exploring thoughts and feelings over the previous 2 weeks. Programme users were presented with items such as “*I've been feeling good about myself* ” and rated themselves on a scale from 1 “None of the time” to 5 “All of the time,” producing a total score between 14 and 70, with suggested cut-offs of <40 (probable depression), 41–44 (possible depression), 45–59 (average mental wellbeing), and >60 (high mental wellbeing).

Perceptions of health were measured using the EuroQol EQ-5D-5L (42), which has five domains focusing on mobility, self-care, usual activities, pain/discomfort, and anxiety/depression, with one question per domain. Each question has five options to choose from ranging from 1 “no problems” to 5 “inability to function,” producing a total score between 5 and 25. An additional EQ-VAS visual analog scale asked how good or bad programme users perceived their health to be, ranging from 0 (the worst health you can imagine) to 100 (the best health you can imagine).

### Statistical analysis

Analysis (led by JS and NH) compared demographics and primary and secondary outcomes for those dropping out or completing, using means (for normally distributed variables) and medians (for variables showing elevated skewness and/or kurtosis >1), with bootstrapped 95% bias adjusted confidence intervals (CI). Following recent guidelines on the interpretation of confidence intervals for independent groups, a mean difference was considered potentially important if the two individual confidence intervals were separated by a gap, indicating a difference at *p* < 0.01 (43, 44). This stricter criterion was adopted due to the number of comparisons being made. The same criterion was then used for the baseline comparison analysis of the standard group with the enhanced group.

The frequency distributions of all outcome measures were explored using graphical methods (boxplots, stem-and-leaf plots), descriptive statistical indices (skewness and kurtosis) as well as through kernel density curve estimation using the R package GGPLOT2. This revealed non-normal distributions (skewed and peaked) with numerous outliers for the primary outcome measures. Consequently, robust statistical methods involving 20% trimmed means and Winzorized variances were used to test hypotheses of change involving pairwise comparisons of time points using the R libraries WRS2 (45) and DescTools. Standard errors and 95% confidence intervals were generated through bias corrected (BAC) bootstrapping and robust standardized mean differences (46) are reported as effect sizes for the weekly METs, sporting minutes, and daily sitting minutes.

Yuen's method for *t*-tests based on trimmed means (45) was used for the primary outcome measures to test altogether five planned comparisons (Null-Hypotheses) of mean change for each outcome measure. These were: baseline vs. 3 months, 3 vs. 6 months, and 6 vs. 12 months. Furthermore, a comparison of baseline vs. 12 months was run to investigate if any washout effects (i.e., a return to baseline values at the end of the programme) were present. Due to considerable data loss at the follow-up time points, the time-specific outcome measures at 3, 6 and 12 months were combined into an average outcome score for each individual as the mean of their valid scores (e.g., a single time point, a combination or any two, or all three). This produced an estimate of the amount of change throughout the follow-up period for each outcome measure, involving the complete sample, without any replacement of missing values. The first planned comparison tested for a change in the means between baseline and the whole follow-up period represented by this computed average. The familywise alpha error was set at 5% for each outcome measure and, accordingly, a Bonferroni adjusted *p*-value of 0.01 was applied to each comparison.

The secondary outcomes were approximately normally distributed, and therefore, paired-samples *t*-tests were used and results are reported in terms of means and standard deviations (SD) as well as Cohen's *d* as effect size index with 95% confidence intervals generated through BAC bootstrapping. The same five planned comparisons were utilized in line with the primary outcome analysis.

Power calculations were conducted to ensure that the available sample sizes for the planned comparisons had sufficient statistical power (>0.85) to reveal small to modest effect sizes *d* that were regarded as meaningful for physical activity interventions (19). Results showed that comparisons based on sample sizes of around *N* = 700 and *N* = 350 had a power of 0.99 and 0.87 respectively to discover a small *d* = 0.20 effect size with alpha set at 0.01, two-tailed. Sample sizes of around *N* = 200 had a power of 0.95 to discover a modest *d* =*0.3*0 with alpha at 0.01, two-tailed. For the smallest available sample size of *N* = 139 the statistical power was 0.82 to discover a *d* =*0.3*0 with alpha at 0.01, two-tailed.

### Ethics

Ethical approval was given by the Faculty of Medicine and Health Sciences Research Ethics Committee at the University of East Anglia (Ref: 20152016-28). Written informed consent was obtained from all participants, who were assured confidentiality, anonymity, and their right to withdraw their data.

## Results

### Protocol deviations

The planned differences between the “standard” and “enhanced” groups in the published protocol (30) were that the enhanced delivery group would have access to optional social support through “exercise buddies” (offered *via* volunteers), and that the physical activity sessions were free and often organized and facilitated by their Get Active Specialist. However, exercise buddies were not recruited at the rate originally planned in the protocol, and therefore were not an option available in the enhanced group. In the two standard delivery areas, by the middle of the 3rd year the programme was running, the Get Active Specialists had begun to put on additional free exercise sessions specifically for the programme users, often run by themselves, offering additional social support and free access to exercise offers. Although these changes from the original protocol diminished the originally anticipated difference in the planned delivery between the two groups, they evolved from participant feedback and demand and reflected the pragmatic nature of this real-world programme delivery and evaluation (28).

Based on deliverer and participant feedback, the ordering and presentation of the questionnaire and booklet were changed, enabling questions to flow more easily. The actual questions and booklet content remained unchanged. Furthermore, to engage more programme users in the evaluation after the initial 12 weeks of activity sessions, “conversation cafes” were hosted by the Get Active Specialists and study team. This presented an opportunity for programme users to socialize at a local venue and enabled the Get Active Specialists to reconnect with them and encourage completion of follow-up questionnaires.

### Comparison of standard vs. enhanced delivery groups at baseline

The baseline data of programme users in each group were examined (see Table 1). Given the similarity in baseline scores, and the contamination of the two delivery groups due to protocol deviations in the real-world delivery, all participants who completed at least one follow-up time point were combined, regardless of allocated group. Whilst this type of decision would be problematic in a controlled research environment, it is common in pragmatic evaluations (47). Data are, therefore, presented based on the sample as a whole for the remaining analysis.

**Table 1:** Baseline characteristics of programme users who completed at least one follow-up questionnaire, by planned delivery group and overall.

	**Standard group**	**Enhanced group**	**Total overall**
	**(*n* = 145–154)**	**(*n* = 560–578)**	**(*n* = 717 with baseline plus one other data point)**
Age^a^	53.86 (51.75–55.84)	57.74 (56.68–58.86)	56.93 (55.91–57.94)
Female N (%)	95 (62)	400 (69)	495 (68)
IMD score^b^	17.42 (16.73–17.63)	15.11 (13.64–16.20)	15.78 (15.11–16.68)
Sitting^a^	427.16 (393.81–465.70)	360.00 (360.00–382.24)	360.00 (360.00–360.00)
MVPA^b^	40.00 (30.00–40.00)	10.00 (0.00–30.00)	20.00 (15.00–20.00)
METS^b^	655.00 (489.92–856.84)	693.00 (693.00–693.00)	678 (594.00–754.00)
Sport^b^	0.00 (0.00–0.00)	0.00 (0.00–0.00)	0.00 (0.00–0.00)
WEMWBS^a^	49.10 (47.48–50.64)	48.60 (47.75–49–49)	48.70 (47.96–49.47)
EQ VAS^a^	56.93 (53.62–60.40)	59.59 (57.93–61.10)	59.04 (57.54–60.52)
EQ-5D-5L^b^	8.89 (8.37–9.41)	8.66 (s8.39–8.93)	8.71 (8.47−8.95)

^a^Mean (95% CIs).

^b^Median (95% CIs).

Female, Frequency (percentage) of female programme participants; IMD, Index of Multiple Deprivation; Sitting (minutes per day); MVPA, Moderate and Vigorous Physical Activity minutes (per week); METS, Metabolic Equivalent of Task (per week); WEMWBS, Warwick-Edinburgh Mental Wellbeing Scale; EQ VAS, EuroQol Visual Analog Scale; EQ-5D-5L, EuroQol five dimensions, five levels. This value represents a weighted health state score based on UK benchmarks.

**Figure 1:**
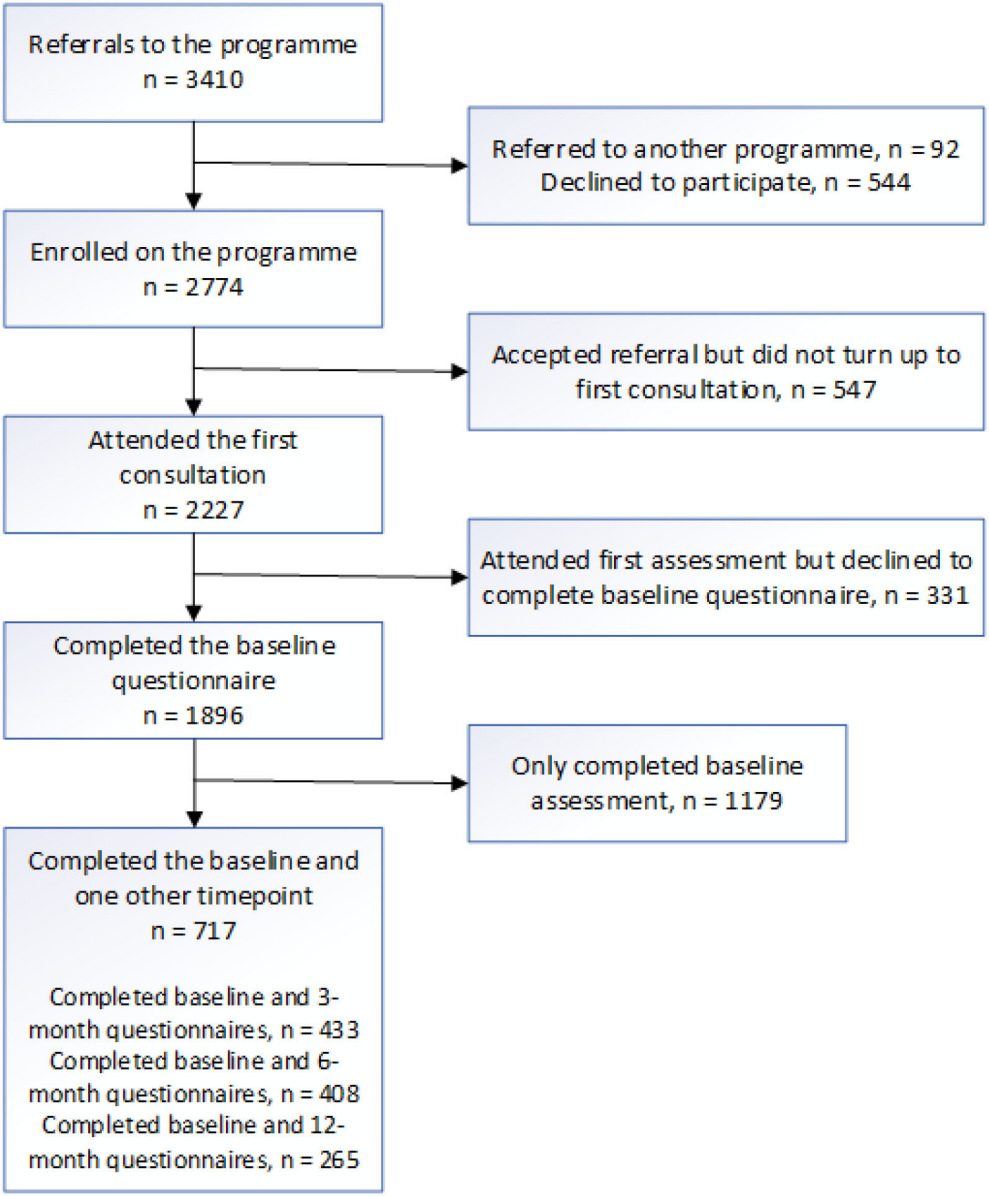
Participant flowchart for referrals, programme enrolment, consultation, evaluation baseline questionnaire, and follow-up questionnaire completion.

The Active Herts programme received 3,410 referrals from four areas of deprivation in the Hertfordshire area. Of these, 1,896 individuals completed baseline questionnaires and 717 (68% female, mean age 56.9 years) at least one follow-up questionnaire (see Figure 1 for full breakdown). Those not completing a baseline questionnaire either (a) did not translate to consultation appointments, (b) were not offered baseline questionnaires (due to the Get Active Specialist prioritizing the consultation over the data collection in cases where, for example, the client was tearful), or (c) declined to be included in the evaluation.

### Attrition from baseline reporting analysis

To assess the amount of bias introduced into the sample by those disengaged with the evaluation after their baseline assessment (evaluation dropouts), their baseline data was compared with those that engaged with the programme evaluation further by completing at least one follow-up time point (completers; see Supplementary Table 2 for full breakdown). This analysis showed that the two groups of programme users were similar on a range of demographic and outcome measures. For the demographic variables the only difference was age, in that completers (*M* = 56.93, 95% CIs 55.89–57.95) were older than evaluation dropouts (*M* = 50.98, CI 50.12–51.80). For primary outcomes, the only difference was that the level of physical activity in completers was slightly higher in terms of MVPA minutes per week at baseline, *Mdn* – 20.00, 95% CIs [15.00–20.00] than evaluation dropouts, *Mdn* −0.00, CI [0.00-0.00], however, both groups were, on average, inactive using Sport England's (2021) criteria (<30 min per week). For secondary outcomes, the only difference was that completers rated their perceived health at baseline as higher, *M* = 59.32, CI [57.87–60.59], than evaluation dropouts, *M* = 53.04, CI [51.60–54.35].

#### Primary outcome measures

##### Weekly METS

Table 2 displays medians and trimmed means for all outcomes. For reported METs, the lower limits of the 95% CIs of the trimmed means at 3, 6, and 12 months were all well above the upper limit of the CI of the trimmed mean at baseline, suggesting a significant gain in the weekly METs after baseline throughout the life of the programme. The spread of the scores (Table 2, winsorized SD, IQR), also increased noticeably between baseline and the follow-up time points indicating much variation in the amount of behavior change in physical activities amongst the participants. At each follow-up time point a substantial data loss occurred due to missed appointments or evaluation dropouts (data loss at: 3 months = 41%, 6 months = 44%, 12 months = 64%). To obtain an overall MET score for the whole follow-up period, an average MET score was calculated across the valid scores at 3, 6, and 12 months for each individual. The mean of these individual averages was *M*_t_ = 2,044, 95% CI [1,892, 2,216]. The results of the five planned comparisons are displayed in Table 3 revealing a substantial increase in the average MET score at 3 months as well as for the whole follow-up period corresponding to effect sizes of practical significance. After 3 months, however, no further gains were made between the 3 and 6, and the 6–12 month periods. At 12 months, the average weekly MET score was still higher (by 1331) in comparison with the baseline, *d* =0.52, CI [0.43, 0.66], providing evidence for maintenance in physical activity behavior change over the life of the programme.

**Table 2:** Descriptive statistics and 95% CIs of outcome measures at baseline, 3, 6, and 12 months.

**Outcome**	**Statistics**	**Baseline**	**3 months**	**6 months**	**12 months**
Weekly METs	*N*	711	418	395	255
	Median (95% CI)	678 (594–749)	1,993 (1,728–2,178)	1,688 (1,478–1,956)	2,093 (1,846–2,294)
	20% trimmed M_t_ (95% CI)	814 (724–913)	2,131 (1,916–2,358)	1,908 (1,678–2,139)	2,249 (1,945–2,572)
	20% winsorized SD	1,624	2,463	2,537	2,798
	IQR	1,484	2,865	2,811	3,083
	Skewness	3.1	1.9	2.0	2.1
Weekly sports minutes	*N*	714	426	403	263
	Median (95% CI)	0 (0–0)	0 (0–30)	0 (0–0)	0 (0–0)
	20% trimmed M_t_ (95% CI)	0 (0–0)	35 (26–43)	20 (13–28)	20 (11–30)
	20% winsorized SD	47	112	90	126
	IQR	0	120	60	60
	Skewness	5.9	4.2	3.1	3.4
Daily sitting minutes	*N*	710	433	406	264
	Median (95% CI)	360 (360–360)	300 (300–300)	300 (300–300)	300 (300–300)
	20% trimmed M_t_ (95% CI)	390 (373–407)	327 (313–345)	317 (297–335)	318 (292–341)
	20% winsorized SD	202	179	178	196
	IQR	300	188	273	240
	Skewness	2.5	1.3	1.1	1.6
EQ VAS perceived health	*N*	717	433	408	265
	Mean (95% CI)	59.0 (57.5–60.5)	66.3 (64.3–68.3)	66.7 (64.69–68.74)	68.0 (65.6–70.5)
	SD	20.4	21.1	20.8	20.5
	Skewness	−0.3	0.3	−0.8	−0.9
EQ-5D-5L health score	N	717	433	408	265
	Mean (95% CI)	8.71 (8.47–8.95)	8.40 (8.09–8.72)	8.69 (8.34–9.03)	8.72 (8.29–9.14)
	SD	3.25	3.34	3.55	3.52
	Skewness	1.1	1.4	1.3	1.23
WEMWBS psychological wellbeing	N	712	430	405	264
	Mean (95% CI)	48.7 (48.0–49.5)	50.3 (49.3–51.3)	50.6 (49.6–51.7)	50.4 (49.1–51.7)
	SD	10.2	10.7	10.4	10.7
	Skewness	−0.4	−0.7	−0.4	−0.5

**Table 3:** Results of the five planned comparisons for all outcome measures.

**Outcome**	**Baseline vs. 3–12 months**	**Baseline vs. 3 months**	**3 vs. 6 months**	**6 vs. 12 months**	**Baseline vs. 12 months**
Weekly METs	*MD*_t_ = 1,234, CI [1,080, 1,389], *p* <0.001, *t* = 15.7, *df* = 414, *d* = 0.60	*MD*_t_ = 1,315, CI, [1,094, 1,536], *p* <0.001, *t* = 11.7, *df* = 244, *d* = 0.56	*MD*_t_ = −207, CI [−577, 164], *N* = 172, NS	*MD*_t_ = 316, CI [−85, 716], *N* = 139, NS	*MD*_t_ = 1,331, CI, [1,032, 1,631], *p* <0.001, *t* = 8.8, *df* = 149, *d* = 0.52
Weekly sports minutes	*MD*_t_ = 26, CI [19,31], *p* <0.001, *t* = 8.4, df = 419, *d* = 0.30	*MD*_t_ = 34, CI, [25,42], *p* <0.001, *t* = 8.0, *df* = 250, *d* = 0.32	*MD*_t_ = −19, CI, [−4, −34], *p* = 0.016, *t* = −2.4, *df* = 108, *d* = −0.11	*MD*_t_ (6 to 12) = −4, CI [−20, 13], *N* = 147, NS	*MD*_t_ = 20, CI, [8, 29], *p* <0.001, *t* = 8.8, *df* = 154, *d* = 0.24
Daily sitting minutes	*MD*_t_ = −64, CI [−80, −48], *p* <0.001, *t* = −7.8, *df* = 420, *d* = −0.32	*MD*_t_ = −71, CI, [−92, −50], *p* <0.001, *t* = −6.7, *df* = 255, *d* = −0.33	*MD*_t_ (3 to 6) = −9, CI [−35, 18], *N* = 187, NS	*MD*_t_ (6 to 12) = 3, CI [−25, 31], *N* = 149, NS	*MD*_t_ = −66, CI, [−94, −38], *p* <0.001, *t* = −6.4, *df* = 154, *d* = −0.36
EQ VAS perceived health	*MD* = 6.64, CI [5.13, 8.14], *p* <0.001, *t* = 8.65, *df* = 706, *d* = 0.33	*MD* = 6.88, CI, [4.99, 8.76], *p* <0.001, *t* = 7.17, *df* = 428, *d* = 0.35	*MD* = 1.58, CI [−0.96, 4.12], *N* = 189, NS	*M* = −0.27, CI [−3.66, 3.12], *N* = 152, NS	*MD* = 6.63, CI, [4.18, 9.09], *p* <0.001, *t* = 5.32, *df* = 262, *d* = 0.33
EQ-5D-5L health score	*MD* = −0.08, CI, [−0.27, 0.11], *p* = 0.40, *t* = −0.85, *df* = 706, NS	*MD* = −0.20, CI, [−0.45, 0.04], *N* = 429, NS	*MD* = 0.39, CI, [0.01, 0.77], *N* = 189, NS	*MD* = 0.19, CI, [−0.20, 0.58], *N* = 152, NS	*MD* = −0.12, CI, [−0.45, 0.21], *N* = 263, NS
WEMWBS psychological wellbeing	*MD* = 1.45, CI [0.63, 2.26], *p* <0.001, *t* = 4.93, *df* = 700, *d* = 0.19	*MD* = 1.45, CI, [0.63, 2.26], *p* = 0.001, *t* = 3.49, *df* = 425, *d* = 0.17	*MD* = −0.14, CI [−1.20, 0.91], *N* = 187, NS	*MD* = −0.86, CI [−2.20, 0.48], *N* = 149, NS	*MD* = 0.97, CI, [−0.08, 2.01], *N* = 260, NS

NS, not significant (*p* >0.01); all CIs are at 95%.

##### Weekly sport minutes (WSM)

As with METs, data loss at each follow-up time point was substantial (data loss at: 3 months = 40%, 6 months = 44%, 12 months = 63%) and an individual average WSM score was calculated across all follow-up points which was *M*_t_ = 26, 95% CI (19, 31). The planned comparisons (Table 3) showed a statistically significant increase from baseline to 3 months by 34 min of weekly sport participation, and by 26 min for the follow-up period. There was a small and only borderline statistically significant reduction in the average duration of sporting activities between 3 and 6 months, and no further change in the means from 6 to 12 months. There was also no indication of a return to baseline values (washout effect) as the average WSM at 12 months was still 20 min above the baseline level.

##### Daily sitting minutes

Table 2 reveals that the lower limit of the CI for the 20% trimmed mean at baseline (373) is not included in the CIs of the means for the repeated measures at 3 (313–345), 6 (297–335), or 12 (292-341) months suggesting a reduction in the average time of reported sitting a day during the follow-up period. This was accompanied by a modest reduction in the spread of the scores after baseline. A considerable data loss occurred at each follow-up time point (data loss at: 3 months = 39%, 6 months = 43%, 12 months = 63%) and an average daily sitting score was calculated for each participant using all valid observations across the follow-up period. The results of the planned comparisons revealed a significant overall reduction of daily reported sitting by 71 min at 3 months, and by 64 min for the whole follow-up period (Table 3). No further reduction in average daily sitting occurred between 3 and 6 months or between 6 and 12 months. The reduction in daily sitting was maintained until the end of the programme as the mean at 12 months was still significantly lower (66 min) compared with the baseline.

#### Secondary outcome measures

##### Perceived health (PH): EQ VAS

PH scores were approximately normally distributed (see Table 2) but data loss at each follow-up time point was substantial (data loss at: 3 months = 41%, 6 months = 44%, 12 months = 64%). An individual's average PH score was therefore calculated across the available scores at 3, 6, and 12 months with a mean of *M* = 65.65, 95% CI [64.15, 67.15]. The planned comparisons (Table 3) suggest a significant yet modest improvement in the average perceived health at 3 months (*d* = 0.35) as well as for the whole follow-up period (*d* =0.33), but no further gains for the periods from 3 to 6 or from 6 to 12 months. The PH mean at 12 months was still significantly higher compared to the PH mean at baseline indicating that the improvement in perceived health at 3 months was maintained until the end of the programme.

##### Health state (HS): EQ-5D-5L

Data loss for perceived health state at each follow-up time point was substantial (data loss at: 3 months = 41%, 6 months = 44%, 12 months = 64%). An average HS score was calculated for each individual across the available data at 3, 6, and 12 months with a mean *M* = 8.65, 95% CI [8.41, 8.90]. None of the planned comparisons reached statistical significance indicating no change of the baseline mean in perceived health state throughout the life of the programme (Table 3).

##### Mental wellbeing (WEMWBS)

Mental wellbeing scores were approximately normally distributed (see Table 2) but data loss at each follow-up time point was substantial (data loss at: 3 months = 41%, 6 months = 45%, 12 months = 64%). As before, an individual average mental wellbeing score was calculated across the available scores at 3, 6, and 12 months. Mean mental wellbeing at baseline was *M* = 50.08, 95% CI [49.30, 50.82] and increased by a small amount (*d* = 0.17) at 3 months. Similarly, the average improvement for the whole follow-up period was small (*d* = 0.19). However, the initial improvements in wellbeing were not maintained over time as there was no significant mean difference between the baseline and 12 months scores (see Table 3).

## Discussion

Overall, attending Active Herts was associated with increased self-reported physical activity and participation in sport, reduced sitting time, increased mental wellbeing, and improved perceptions of health. The average increase in physical activity was clinically meaningful (sustained average increase of 1331 weekly METs), with over 1200 METs representing a change of 150 min of vigorous intensity, 300 min of moderate intensity, or over 350 walking minutes per week over the duration of the programme. This change highlights the success of the programme and exceeds the recommended weekly physical activity level for health benefit (2).

Part of the improvement in physical activity was due to an increase in reported weekly sporting minutes of between 20 and 26 min, which on its own would almost reach the Sport England (4) threshold for being fairly active (30–149 min). A significant reduction in reported sitting of over 1 hour per day was also seen, which would offer additional benefit over and above being regularly active (2, 48). These effects are likely to be due to the intensive first 3 months of the Active Herts programme, which offered free or subsidized access to structured exercise and sporting classes, which were then recommended for a further 9 months, with ongoing access to exercise opportunities at a small cost to the programme user. This access, alongside conversation cafes, further offered additional peer and social support (28). It must be noted at this point, however, that measured improvements in physical activity and sitting are based on self-reported data, which may be at risk of social desirability bias and over-reporting (49).

This programme harnessed the evidence-base around the COM-B model for both increasing physical activity (17) and reducing sitting (18). The Get Active Specialists were trained to perform a COM-B behavioral diagnosis to assess the programme user's barriers and facilitators to behavior change, looking for COM-B “missing links” (barriers) and “hooks” (reasons to change and facilitators). This was performed using motivational interviewing (23, 36, 37) and health coaching (26). Following assessment and formulation, the Get Active Specialists were able to deliver the BCTs included in the programme protocol that have been scientifically shown to lead to greater success in physical activity behavior change interventions (19), tailored to the programme user's needs. To our knowledge, this is the first real world physical activity programme to bring these elements of the Behavior Change Wheel, COM-B model (15, 16), Motivational Interviewing (23, 36, 37), and Health Coaching (26, 50) together in this way and to test them in a pragmatic evaluation.

Active Herts further enhanced programme user's perception of their overall health. This improvement was seen at the first follow-up at 3-months and was maintained throughout the 12-month duration of the programme, although the effect was overall modest in size. Mental wellbeing showed a small, significant improvement at 3 months; and although this increase from baseline to the end of the programme continued to trend, it was not found to be statistically significant. This may be partly explained by participants baseline mental wellbeing (WEMWBS) scores already sitting in the “average mental wellbeing” category (41). There was also no significant change in the health state score, suggesting the programme did not have an impact upon combined and weighted scores for mobility, self-care, usual activities, pain/discomfort, and anxiety/depression. This programme was aimed at individuals identified as at risk of CVD and mild to moderate mental health concerns, and as such, they may have a multitude of reasons for this lack of change in health state score that would warrant further investigation.

### Strengths

This study had a number of strengths which included having the methods pre-registered in a detailed protocol (30), the content being heavily guided by BCTs found to be present in effective interventions (19) and the delivery based on theoretical and methodological approaches from health psychology and behavioral science (22, 35, 38). The evaluation in a real-world setting, as opposed to a controlled research trial environment, also provides evidence that is realistic to routine practice. The ongoing training and supervision of the Get Active Specialists from a HCPC (Health and Care Professions Council) Registered Practitioner Health Psychologist (AC) throughout the programme also ensured the fidelity of the approach, allowing correction and reinforcement of early skill development, and maintenance of the approach over time. A further strength was the large sample size allowing the ability to produce precise effect size estimates as indicated by narrow confidence intervals.

There was a good buy-in from GPs to refer their patients in to the Active Herts programme. Following the end of the original funding period of the programme, funding has been acquired to continue the service, such as the re-branded Active Watford and Three Rivers programme, funded by the Premier League, and Broxbourne's continued use of the Active Herts programme, funded by local authority means. Other local authorities and Active Partnerships have received training in the approach (32) and future initiatives should consider the evidence for sustained behavior change presented here. To capitalize on this approach, allocated funding is needed nationally, to maintain roles such as the Get Active Specialist within the healthcare system, with adequate access to training and supervision. This would also align with GPs preference to refer to a qualified exercise professional within a multidisciplinary team (51) and widen opportunities for real-world research of this kind.

### Limitations

The Active Herts programme was originally planned to have two different forms of delivery, the enhanced delivery model with access to free exercise sessions for 12 weeks and exercise buddies, and the standard delivery model with signposting to subsidized exercise sessions and no exercise buddies. However, changes during the programme, based on pragmatic responses to participant demand and deliverer feedback, led to almost identical approaches being adopted in both groups (free activity sessions and no exercise buddies). These approaches were further contaminated by combined training and supervision/booster sessions. Without major differences found between the two groups, the sample was treated as one, ruling out a comparison of the two different modalities. Furthermore, without a comparative intervention or a suitable control group, whether active or passive, the evidence for the effectiveness of the specific BCTs employed by Active Herts for inactive adults remains preliminary.

Given the form of data collection was self-report, over- or under-reporting or recall biases due to social desirability or misremembering may have also occurred, which should be taken into consideration when interpreting the results. While objective behavioral measures could counteract this limitation, there was no resource for this in the current evaluation, due to its real-world nature. The issue of attrition rates in the evaluation was also a considerable limitation with over 40% at 3 and 6 months, and over 60% at 12 months, even in a sample that completed baseline measures and attended the consultations. Therefore, the results may over-represent the experience of a select sample of more motivated programme users that were different from those who did not participate in the evaluation or dropped out from the programme all together. Future work needs to explore methods of maintaining deliverer and programme user engagement with evaluation procedures for the whole duration of the programme to ensure data capture at each time point is achieved.

This study provided an evaluation of the programme outcomes only. Future research should address questions relating to the mechanism of action for behavior change from inactive to active. It should also examine the relevance of specific BCTs employed by Active Herts in facilitating this process successfully for maintenance after the programme has ended. It was also not practical or affordable within the programme structure or budget to collect objective outcome measures related to CVD risk, such as body mass index (obesity), blood pressure (hypertension) or glucose control (diabetes). Future research should extend outcome measures to such variables, with considerations of how they would be measured in clinical practice.

## Conclusion

This evaluation provides evidence that an intervention based on health psychology and behavioral science, which utilizes methods from previous research of what works, can be delivered in a real-world setting, and lead to significant and sustainable improvements in physical activity, sedentary behavior, and perceived health, as well as initial improvements in mental wellbeing. This was an exploratory study of a “real-world” programme embedded in routine delivery, and therefore, a pragmatic approach to data analysis was taken. This extends previous research in this area and offers a template for future interventions to build upon. To utilize this approach in a healthcare setting, exercise professionals should be embedded in the healthcare system. This will require suitable and sustainable funding for positions, training and supervision. Furthermore, evaluation should be considered from the onset, to ensure fidelity of programme delivery, and efficacy in relation to behavior change, health and wellbeing outcomes. The full Active Herts package can be found on the Local Government Association website (32) and has been promoted as a case study for best practice.

## Data availability statement

The raw data supporting the conclusions of this article will be made available by the authors, without undue reservation.

## Ethics statement

The studies involving human participants were reviewed and approved by the Faculty of Medicine and Health Sciences Research Ethics Committee at the University of East Anglia (Ref: 20152016-28). The patients/participants provided their written informed consent to participate in this study.

## Author contributions

AC, NH, and AJ contributed to the conception and design of the work. AC, NH, AJ, and DK contributed to the acquisition of the data. JS, NH, AJ, AB, SC, and AC contributed to the analysis and interpretation of data. AC, JS and NH wrote the first draft of the manuscript. All authors contributed to manuscript revision, read, and approved the submitted version.

## Funding

This work was supported by funding from Sport England (Ref: 2015000295), Broxbourne Borough Council, East and North Herts CCG, Herts Valley CCG, Hertfordshire Public Health, Herts Mind Network, Mind in Mid Herts, Herts Sports Partnership, and time allocation from the University of Hertfordshire, University College London and the University of Bedfordshire. Open access fees have been provided by the Institute for Sport and Physical Activity Research (ISPAR), University of Bedfordshire.

## Conflict of interest

The authors declare that the research was conducted in the absence of any commercial or financial relationships that could be construed as a potential conflict of interest.

## Publisher's note

All claims expressed in this article are solely those of the authors and do not necessarily represent those of their affiliated organizations, or those of the publisher, the editors and the reviewers. Any product that may be evaluated in this article, or claim that may be made by its manufacturer, is not guaranteed or endorsed by the publisher.
