# Supplementary material for: Outcome evaluation of Active Herts: A community-based physical activity programme for inactive adults at risk of cardiovascular disease and/or low mental wellbeing
Source: Front Public Health. 2022 Sep 9;10:903109. doi: 10.3389/fpubh.2022.903109 (PMC9500394; doi:10.3389/fpubh.2022.903109)
Supplement: Supplementary file 1 [file Data_Sheet_1.docx]

**Supplementary Table 1: Programme content specified by behaviour change techniques and linked to constructs of the COM-B model**

| **Programme component** | **COM-B construct targeted** | **Behaviour change technique** | **Content** |
| --- | --- | --- | --- |
| Booklet (both groups) | Reflective motivation | 9.2. Pros and Cons | A page asking whether exercise is good for you and programme users are given two blank columns to fill out with possible advantages and disadvantages of becoming more active. They are then asked how confident they feel about becoming active on a scale of 1-10. |
|  | Psychological capability;* Reflective motivation | 1.2. Problem Solving | Programme users are asked to think about their current situation and to list the things that might be currently stopping them from being active and how they might overcome them.  In contrast to the problem solving page which focuses on current problems, another page explains how even the most habitual exercisers can struggle at times. Programme users are asked to think about situations in the future that may affect their progress and then about options to avoid or cope with these situations using an If-Then (implementation intention) planning technique. |
|  | Reflective motivation | 1.1. Goal setting (behaviour) | Programme users are given the opportunity to set short (two weeks), medium (3 months), or long-term (12 months) goals, and then rate how confident they are of achieving each one from 1-10. |
|  | Psychological capability; Reflective motivation | 1.4. Action planning | A page allowing programme users to complete sections referring to their plans to becoming more active in terms of what they are going to do, where they are going to do it, when they are going to do it, and who they are going to do it with. A second page allows them to explore their behavioural regulation and time management by mapping out the week in terms of morning, afternoons, and evenings. |
|  | Psychological capability; Reflective motivation | 2.3. Self-monitoring of behaviour;  *5.4. Monitoring of emotional consequences*** | Programme users are given an exercise and activity diary to track their progress and highlight their engagement. A table contains columns for the date, activities completed, time in minutes, enjoyment level (from 1, low to 10, high), and how they felt after completing the activity. The table contains several rows so programme users can track this over time. |
|  | Psychological capability; Reflective motivation | 5.1. Information about health consequences;  5.6. Information about emotional consequences | A page summarises the health and emotional benefits of being active in a positively framed manner. For example, did you know that being active can ‘help you manage high blood pressure’ and ‘make you feel good and improve your mental health’. |
|  | Psychological capability* | 4.1. Instruction on how to perform the behaviour | Programme users are given the national exercise guidelines for moderate and vigorous physical activity. Additionally information is given for examples of moderate and vigorous physical activity, how to break up long periods of sitting, how to improve balance to reduce the chance of falls, and an example of how these activities can fit into everyday life. |
|  | Automatic motivation | 10.9. Self-reward | Programme users are told the importance of rewarding themselves for the effort they make towards their activity goals. Examples are then given of how to reward themselves in ways that are healthy and free. For example, ‘listen to music’ or ‘have a nice relaxing bath’. Self-reward is also discussed briefly during the goal setting page when thinking about what success looks like. |
|  | Social opportunity | *5.3. Information about social and environmental consequences*** | Images of people engaging in physical activity were presented with messaging such as ‘I’m doing it to meet new people’. |
|  |  |  |  |
| Consultation (both groups) | Social opportunity; Automatic motivation | 3.1. Social support (unspecified);  3.2. Social support (practical)  3.3. Social support (emotional)  *4.2. Information about antecedents*** | Programme users are given an initial 45 minute one-to-one consultation in person where motivational interviewing and health coaching are used to structure and tailor the session to the programme user’s needs.  A COM-B analysis is performed to address barriers and facilitators to physical activity, identifying factors that may prevent physical activity performance, with the specialist recognising these as ‘missing links’ in the COM-B system.  Desire for change is discussed as a ‘hook’ to support the programme user towards becoming more active. Get Active Specialists signpost and support the booking of activities and discuss goals and plans, while providing emotional support.  This is then repeated in subsequent consultation meetings at 3, 6, and 12 months. The additional consultations will vary between 15-30 minutes and are optional based on participant needs. |
|  | Social opportunity*; Automatic motivation | 9.1. Credible source | Get Active Specialists who are expert and registered exercise professionals, trained in motivational interviewing, health coaching and behaviour change, with specialist knowledge of obesity, diabetes, exercise referral, and mental health discuss becoming more active in a favourable light with programme users. |
|  | Reflective motivation | 15.1. Verbal persuasion about capability | Programme users will set goals and the Get Active Specialists will encourage the belief in their ability to fulfil those goals and make long-term change. |
|  |  |  |  |
|  | Reflective motivation | 15.3. Focus of past success | During the consultation programme users will set physical activity goals and the Get Active Specialists will discuss previous success or progress. |
|  |  |  |  |
| Exercise sessions (both groups) | Psychological capability; Social opportunity;  Physical opportunity | 4.1. Instruction on how to perform the behaviour;  6.1. Demonstration of the behaviour;  8.1. Behavioural practice/rehearsal  *8.6. Generalisation of target behaviour*** | Programme users have the opportunity to attend 12 weeks of exercise classes either referred to them (standard delivery) or organised as bespoke sessions (enhanced delivery) by the Get Active Specialists. These involve detailed instruction on how to perform a range of exercises (e.g. yoga, Pilates, light to moderate-intensity circuit training). During these classes programme users are given demonstrations of the correct way to perform the activities and provided with ample opportunity to practice and gain confidence in performing the exercises. They are further encouraged to perform these activities between sessions at home. |
|  | Physical capability | 8.7. Graded tasks | During the exercise classes, exercise specialists will encourage programme users to start slowly and build up intensity throughout the 12 weeks. |
|  |  |  |  |
| Booster call (both groups) | Social opportunity; Reflective motivation; | 3.1. Social support unspecified;  15.1. Verbal persuasion about capability;  7.1. Prompts and cues | Programme users receive a phone call at 2 weeks, which is approximately 5 minutes in duration prompting them to keep working towards their physical activity goals and stating that they are capable of achieving them. |
|  |  |  |  |
| Test messages (both groups) | Social opportunity; Reflective motivation; | 3.1. Social support unspecified;  15.1. Verbal persuasion about capability;  7.1. Prompts and cues | A text message is sent to programme users at 2, 6, and 12 weeks prompting them to keep working towards their physical activity goals and stating that they are capable of achieving them. |
|  |  |  |  |
| Exercise buddies and tailored exercise classes (enhanced delivery only) | Social opportunity; Automatic motivation | 3.2. Social support (practical)  3.3. Social support (emotional) | For programme users in the enhanced delivery areas, Get Active Specialists will also run and/or organise a range of exercise classes based on the preferences of programme users, where they may also be paired with an exercise buddy to help them attend the exercise classes and provide emotional support if needed. |

Note: *denotes that a BCT was not explicitly linked to a COM-B construct in the consensus study from Cane et al. (2015), but the authors believe this BCT will impact this COM-B construct. ***not included as a code in the original protocol but present in the intervention*

**Supplementary Table 2: Dropout analysis comparing key demographic, primary, and secondary outcome measures between programme users who only completed baseline (dropouts) vs those that completed baseline and at least one other time-point (completers). Medians were used when skewness and kurtosis statistics suggested non-normally distributed data.**

| **Measure** | **Complete** | **Sample size** | **Mean/Median (95% bias**  **corrected CIs)** | **SD** |
| --- | --- | --- | --- | --- |
| IMD^b^ | Dropout | 1155 | 17.46 (16.72-17.76) |  |
|  | Completers | 726 | 15.78 (15.11-16.73) |  |
| Age at referral^a^ | Dropout | 1164 | **50.98 (50.12-51.80)** | 15.13 |
|  | Completers | 732 | **56.93 (55.89-57.95)** | 13.97 |
| Female | Dropout | 1164 | 846 (73) |  |
|  | Completers | 732 | 495 (68) |  |
| Weekly METs^b^ | Dropout | 1110 | 693.00 (693.00-693.00) |  |
|  | Completers | 711 | 678.00 (594.00-754.00) |  |
| MVPA minutes per week^b^ | Dropout | 1118 | **.00 (.00-.00)** |  |
|  | Completers | 715 | **20.00 (15.00-20.00)** |  |
| Sporting minutes per week^b^ | Dropout | 1134 | .00 (.00-.00) |  |
|  | Completers | 714 | .00 (.00-.00) |  |
| Sitting minutes per day^b^ | Dropout | 1123 | 360.00 (360.00-360.00) |  |
|  | Completers | 710 | 360.00 (360.00-360.00) |  |
| EQ VAS^a^ | Dropout | 1120 | **53.14 (51.81-54.50)** | 23.42 |
|  | Completers | 717 | **59.04 (57.66-60.36)** | 20.42 |
| EQ-5D-5L^b^ | Dropout | 1120 | 8.89 (8.70-9.08) | 3.45 |
|  | Completers | 717 | 8.71 (8.46-8.95) | 3.25 |
| WEMWBS^a^ | Dropout | 1091 | 47.29 (46.60-48.01) | 11.46 |
|  | Completers | 717 | 48.70 (47.93-49.48) | 10.19 |

Note: Note: ^a^Mean (95% CIs); ^b^Median (95% CIs). Female – Frequency (percentage) of female programme participants; IMD – Index of Multiple Deprivation; Sitting (minutes per day); MVPA – Moderate and Vigorous Physical Activity minutes (per week); METS – Metabolic Equivalent of Task (per week); WEMWBS - Warwick-Edinburgh Mental Wellbeing Scale; EQ VAS – EuroQol Visual Analogue Scale; EQ-5D-5L – EuroQol five dimensions, five levels. This value represents a weighted health state score based on UK benchmarks. Bold font signifies that confidence intervals do not overlap, indicating a difference at *p* < .01.
